# miR-4461 Regulates the Proliferation and Metastasis of Ovarian Cancer Cells and Cisplatin Resistance

**DOI:** 10.3389/fonc.2021.614035

**Published:** 2021-03-09

**Authors:** Lei Dou, Yi Zhang

**Affiliations:** Department of Gynecology, The First Affiliated Hospital of China Medical University, Shenyang, China

**Keywords:** cisplatin, progression, PTEN, ovarian cancer, miR-4461

## Abstract

microRNAs (miRNAs) are of great significance in cancer treatment, which may have a desirable result on the regulation of tumorigenesis, progression, recurrence, and chemo-resistance of ovarian cancer. However, the research on the further potential application of miR-4461 in ovarian cancer is little and limited. Therefore, the study in this paper focus on the investigation of the of miR-4461 in ovarian cancer progression and chemo-resistance. The phenomenon that the proliferation and metastasis of ovarian cancer cells can be promoted by miR4461 is revealed in functional assays. Through the bioinformatics and luciferase reporter analysis, the PTEN is validated to be the direct target of miR-4461 in ovarian. The association between the expression of miR-4461 and PTEN is negative in in human ovarian cancer tissues. The distinction of growth and metastasis capacity between miR-4461 knockdown ovarian cancer cells and control cells is partially abolished by si-PTEN. Moreover, it was found that cisplatin treatment has obvious effect on the miR-4461 knockdown ovarian cancer cells. In summary, the data given in this paper indicate that the miR-4461 can be regarded as a potential onco-miRNA in ovarian cancer by targeting PTEN.

## Introduction

Ovarian cancer (OC) has been one of the most global malignant tumors among the women (1). The tendency of the incidence of the OC in the US is descend. However, on the contrary, it is rising in China (2). Generally, OC patients do not usually experience noticeable symptoms in the early stage of tumorigenesis and until now, there is no efficacious method which has been found for the early detection and treatment of OC. As a result, most OC patients have not been diagnosed with ovarian cancer until advanced stage, and they miss the best opportunity for treatment (3, 4). More than 70% advanced OC patients will recur after surgery (5). The therapy based on th Cisplatin is one of the first-line treatment for OC patients, while most patients develop resistance after a period of medication (6–8). Therefore, it's pressing to explore the potential mechanism of OC and uncover new treatment target to improve the prognosis of OC patients.

MicroRNAs (miRNAs) with about 22 nucleotides in length exceptionally expressed in cancers as reported (9). In addition, miRNAs may take part in the regulation of multiple steps so that it may become promising treatment targets for OC. For instance, miR-193b-3p is available for anticancer function in ovarian carcinoma cells by targeting p21-activated kinase 3 (9). MiR-338-3p inhibits OC cells proliferation and metastasis by Wnt/catenin beta and MEK/ERK signaling pathways (10). Crucially, increasingly evidence has emerged to emphasize that the function of miRNAs in cisplatin resistance in human cancers. For instance, the sensitivity on the cisplatin of miR-654-3p is enhanced by targeting QPRT and the PI3K/AKT signaling pathway in OC cells is suppressed (11). MiR-186 achieved the regulation of cisplatin sensitivity on ovarian cancer cells in two directions simultaneously, which not only inhibiting PIK3R3 and PTEN, but also promoting expression of APAF1 as well (12). MiR-4461 is a new-found miRNA, of which the function and mechanism of action in biological processes and diseases have not been yet fully investigated. The conclusion that miR-4461 suppresses tumorigenesis of renal cell carcinoma is given in the previous research (13). Moreover, the miR-4461 which originated from the bone marrow mesenchymal stem cell exosomes suppresses tumorigenesis by reducing COPB2 expression in colorectal cancer (14). Whereas, the function of miR-4461 in OC was unclear.

In recent studies, the suppression on proliferation and metastasis of ovarian cancer cells by miR-4461 has been demonstrated by biological function. It was revealed that PTEN was one of the direct targets of miR-4461 in ovarian cancer cells by the further deep mechanism research. What's more, miR-4461 knockdown OC cells show much more sensitiveness on cisplatin treatment. Based on the research above, it is highlighted that the miR-4461 is of great significance in regulating the progression of OC and cisplatin resistance.

## Materials and Methods

### Collection of Human OC Clinical Tissue Specimens

Human OC tissues were obtained from 40 OC patients from the First Affiliated Hospital of China Medical University (Shenyang, Liaoning, China). All the patients have signed the informed consent about tissue donation for research purpose before surgery. The research design was estimated and endorsed by the Clinical Research Ethics Committees of the First Affiliated Hospital of China Medical University, and whole experimental approach were put into effect in conformity to the guidelines of the Declaration of Helsinki.

### Cell Lines and Cell Culture

HO8910 and A2780, two human ovarian carcinoma cell lines were acquired from the American Type Culture Collection (Manassas, VA). Normal ovarian surface epithelial cells (OSE) cells were provided by Nelly Auersperg (University of British Columbia, Vancouver, Canada). Two ovarian carcinoma kinds cells were cultured in RPMI-1640 medium (Solarbio, Beijing, China), which contains 10% fetal bovine serum (Thermo Fisher, Wilmington, DE, USA), 1% penicillin-streptomycin solution (Procell, Wuhan, China) with 5% concentration of CO2 at 37°C. Normal OSE cells were cultured in medium 199/MCDB 105 medium with 10% FCS.

Dissociated with 0.5% trypsin, HO8910, and A2780 cells were planted into six-well plates. Infected by the miR-4461 sponge virus or miR-4461 mimic virus and their control virus, steady infectants were screened by using puromycin as adopted in previous reports (15).

HO8910 and A2780 cells were transfected with si-PTEN and control siRNA. The siRNAs were transfected into the hepatoma cells at a final concentration of 200 nM using lipofectamine 2000 Transfection Reagen according to the manufacturer's instructions (Thermo Fisher Scientific). The cells were harvested or subjected to further downstream experiments 24–72 h after transfection. Gene knockdown was validated by western blotting. The material involved in this research, like miR-4461 sponge lentivirus, miR-4461 mimic lentivirus, and PTEN siRNA were all purchased from Shanghai GenePharma (Shanghai, China).

### Cell Proliferation Assays

In the assay of the CCK8, HO8910 miR-4461 sponge or A2780 cells miR-4461 sponge and their control cells were planted into 96-well plates (3 × 10^3^ cells per well). By using Kit-8, a Cell Counting, the ATP activity was measured at the specified time. The scheme of the research was given as following: Each well was injected 100 μl the cell suspension with 10 μl CCK-8 solution in a 96-well plate. After then, the plate needs to be incubated in the incubator for 1 h. At last, the the absorbance was quantified at 450 nm by a microplate reader (Synergy H1; BioTek Instruments, Inc., Winooski, VT, USA) (16).

### Colony Formation Assays

For the assay of colony formation, HO8910 miR-4461 sponge or A2780 cells miR-4461 sponge and their control cells were planted into 12-well plates with 3 × 10^3^ cells each well, which get cultured in the circumstance of 37°C for 7 days and then fixed with 10% neutral formalin for more than 4 h. After dying with crystal violet (Beyotime, Haimen, China), the photos of the cells and the results were produced by a microscope (Olympus, Tokyo, Japan).

### EdU Immunofluorescence Assays

HO8910 miR-4461 sponge or A2780 cells miR-4461 sponge and their control cells were planted into 96-well plates and tested by the EdU Kit (RiboBio) at 48 h in order to satisfy the staining of cell EdU immunofluorescence, according to the manufacturer's protocol. Moreover, the results were measured and analyzed by adopting a Zeiss axiophot photomicroscope (Carl Zeiss) as hardware and Image-Pro plus 6.0 as software.

### Cell Cycle Assays

HO8910 miR-4461 sponge or A2780 cells miR-4461 sponge and their control cells were planted into 6-well plates for 48 h. Using trypsin to digest cells firstly and then centrifugation. Wash cell pellet with ice-cold PBS twice. Centrifugate the cells 600 g for 5 min and transfer the tube to ice. Slowly resuspend the cells with ice-cold 70% ethanol in distilled water. Place cells at −20°C before staining and analysis. Centrifugate the cells 1000 g for 5 min at 4°C. Remove the ethanol and resuspend cells in 1 ml ice-cold PBS. Centrifugate the cells 500 g for 10 min at 4°C and remove PBS. Then rewashed with 1 ml ice-cold PBS for twice. Then the cells were stained with Propidium Iodide (PI) (40 mg/ml, Abbkine, Inc, China) and RNase A (250 mg/ml, Roche Diagnostics) for 30 min at 37°C in dark. Data were collected using a Molflo XDP (Beckman Coulter, Inc.250 S.Kraemer Boulevard Brea, CA 92821, USA) equipped with a Spectraphysics argon ion laser and analyzed using Summit (Beckman Coulter, Inc.250 S. Kraemer Boulevard Brea, CA 92821, USA). Results represent a minimum of 20,000 cells assayed for each sample.

### Wound Healing Assay

For wound healing assay, HO8910 miR-4461 sponge or A2780 cells miR-4461 sponge and their control cells were planted into six-well plates. a plastic pipette tip was utilized to make a wound on the monolayer of the cells. After repeated resins with medium, the cast-off cells are removed and the results can be photographed by microscope at 0 and 36 h.

### Cell Migration Assays

As for cell migration experiments, 2 × 10^5^ HO8910 miR-4461 sponge or A2780 cells miR-4461 sponge and corresponding control cells were planted into the upper chamber of a polycarbonate transwell in serum-free RPMI-1640 medium. In the lower chamber of the polycarbonate transwell, RPIM-1640 medium which contained 20% FBS is added into as chemo-attractant. The chamber of the transwell is fixed by 10% neutral formalin for over 4 h. All the sponge and control cells were incubating for 24 h and then were dyed with crystal violet (Beyotime), which were quantified by microscope (Olympus). The average number of the cells in each field is adopted as the representation of the cell number.

### Cell Invasion Assays

Similar with the cell migration assays, the serum-free DMEM medium is adopted in the cell invasion experiment, 2 × 10^5^ HO8910 miR-4461 sponge or A2780 cells miR-4461 sponge were seeded into the upper chamber of a polycarbonate transwell. And the lower chamber of the transwell is filled with the chemo-attractant, the RPIM-1640 medium with 20%FBS contained. The chamber of the transwell is fixed by 10% neutral formalin for over 4 h. However, different with the cell migration assays, all the sponge and control cells were incubating for 36 h and then were dyed with crystal violet (Beyotime), which were quantified by microscope (Olympus). The average number of the cells in each field is adopted as the representation of the cell number.

### Luciferase Reporter Assays

The cDNA fragment of PTEN 3′-UTR was inserted into luciferase reporter plasmid in which there are wild-type or mutant miR-4461 binding site (Promega, Madison, WI). Simple, OC cells were co-transfected with miR-4461 sponge, miR-4461 mimic, and miR-control by siRNA transfection. Moreover, the pMIR-reporter luciferase vector which contained specific sequence of wild-type or mutant PTEN fragment is also applied (Invitrogen, NY, USA). After the transection, all the cells were collected and lysed for a 48 h detection. By the adopting of the Renilla luciferase activity, the luciferase activity was normalized (17).

### Real-Time PCR

HO8910 miR-4461 sponge or A2780 cells miR-4461 sponge and their control cells were planted into six-well plates for 48 h. Then total RNA from cells or tissues was extracted with the TRIzol reagent (Takara). SYBR PrimeScript TM miRNA RT-PCR Kit (TaKaRa Bio Group, Shiga, Japan) was used for miRNA reverse transcription and miRNA expression measuring. U6 RNA was used as the internal control for miRNA. The total mRNA was synthesized into cDNA by ThermoScript TM RT-PCR system (Invitrogen, 11146-057), and mRNA expression was measured by RT-PCR using the ABI PRISM 7300 sequence detector (Applied Biosystems). PCR conditions included 1 cycle at 95°C for 5 min, followed by up to 40 cycles of 95°C for 15 s (denaturation), 60°C for 30 s (annealing) and 72°C for 30 s (extension). β-actin was used as the internal reference for mRNA. The PTEN primer sequences were as follow: 5′ TCCCAGACATGACAGCCATC 3′, reverse: 5′ TGCTTTGAATCCAAAAACCTTACT 3′. The β-actin primer sequences were as follow: 5′ GGCCCAGAATGCAGTTCGCCTT 3′, reverse: 5′ AATGGCACCCTGCTCACGCA 3′. All data were normalized to the internal controls, and fold changes were calculated by the specific quantification method (2^−ΔΔCT^).

### Western Blotting Assays

HO8910 miR-4461 sponge or A2780 cells miR-4461 sponge and their control cells were planted into six-well plates for 48 h. Cells were immersed in RIPA cell lysate containing protease inhibitor for 30 min, and the supernatant was acquired after centrifugation. Total protein concentration was measured using the BCA kit (Pierce; Thermo Fisher Scientific, Inc.) according to the manufacturer's protocol. After electrophoresis, the protein sample was transferred to a PVDF membrane, blocking with the 5% skim milk. Rabbit antihuman PTEN antibody (Proteintech, Chicago, USA), PARP antibody (Proteintech, Chicago, USA), or mouse anti-human GAPDH antibody (Proteintech, Chicago, USA) were added separately and incubated overnight at 4°C. membranes were incubated with secondary antibody at room temperature for 2 h. Chemiluminescent signals were detected by the ECL Kit and b-Actin was used as internal control.

### Apoptosis Assay

HO8910 miR-4461 sponge or A2780 cells miR-4461 sponge and their control cells were seeded into six-well plate and then treated with cisplatin (4 μg/ml) for 48 h, followed by staining with Annexin V and 7-AAD for 15 min at room temperature in the dark. Apoptotic cells were determined by an Annexin VFITC Apoptosis Detection Kit I (BD Pharmingen, San Diego, CA) and detected by flow cytometry according to the manufacturer's instructions.

### Statistical Analysis

All statistical analyses were performed using GraphPad Prism (GraphPad Software, Inc. La Jolla, USA). Groups of data were compared by using *t*-tests or Bonferroni Multiple Comparisons Test (*p < 0.05). *p*-values <0.05 was considered statistically significant.

## Results

### miR-4461 Facilitated Ovarian Cancer Cells Proliferation *in vitro*

**Figure 1:**
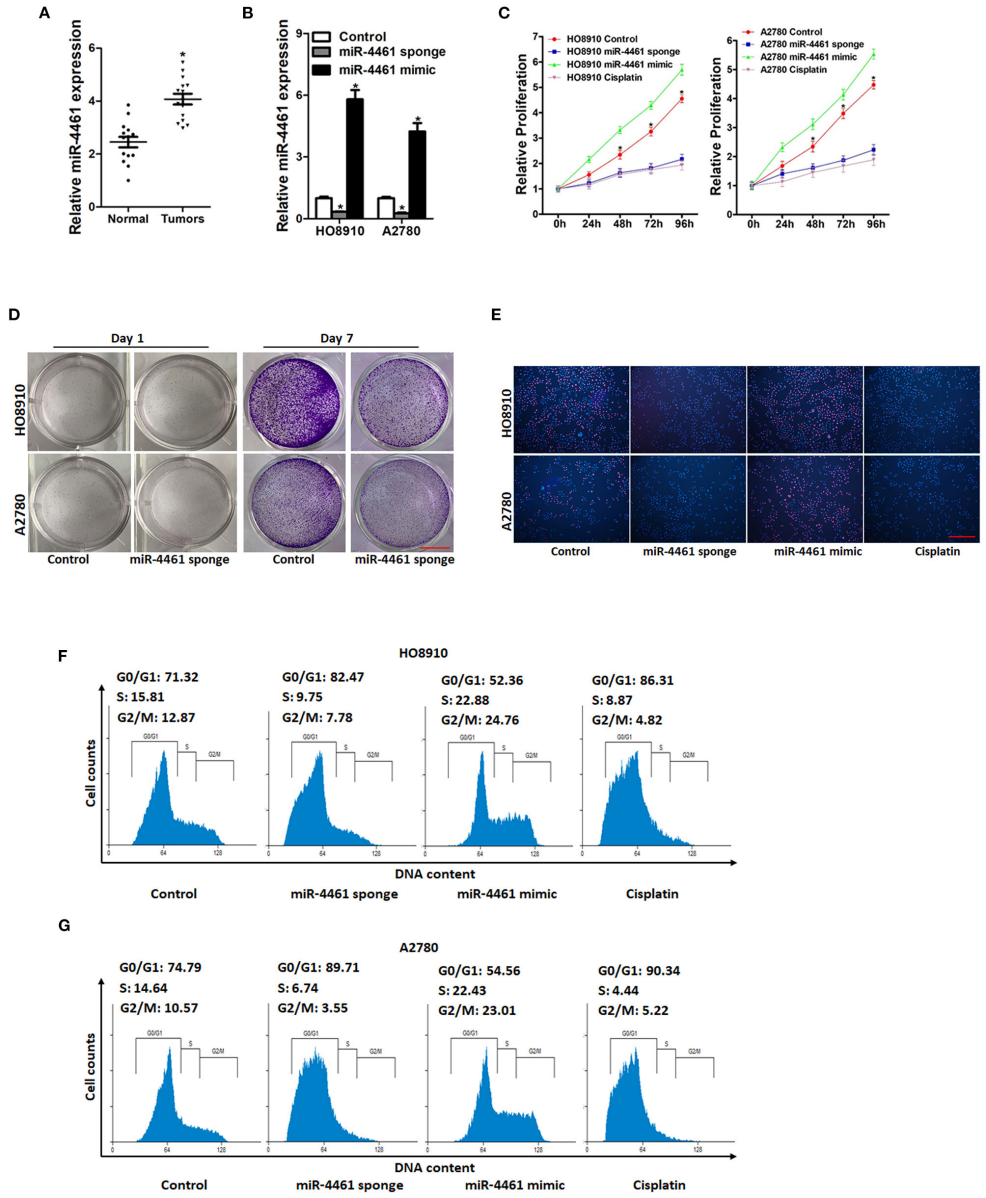
miR-4461 knockdown inhibits ovarian cancer cells proliferation. **(A)** Real-time PCR analysis miR-4461 levels in OC tissues and normal tissues. **(B)** The knockdown and overexpression effect of miR-4461 in HO8910 and A2780 cells was detected by real-time PCR analysis. **(C)** Cell proliferation in miR-4461 sponge or miR-4461 mimic and their control cells was quantified by using CCK-8 assays. Cisplatin (1 μg/ml) used as a positive control. **(D)** Colony formation assays of HO8910 miR-4461 sponge or A2780 miR-4461 sponge and their control cells. Scale bar = 100 μm. **(E)** Representative images of EdU staining of proliferating miR-4461 sponge or miR-4461 mimic and their control cells. EdU+ cells were stained with red immunofluorescence. The nuclei were counterstained with DAPI (blue). Cisplatin (1 μg/ml) used as a positive control. Scale bar = 50 μm. **(F)** Cell cycle in HO8910 miR-4461 sponge or miR-4461 mimic and their control cells was assessed by flow cytometry. Cisplatin (1 μg/ml) used as a positive control. **(G)** Cell cycle in A2780 miR-4461 sponge or miR-4461 mimic and their control cells was assessed by flow cytometry. Cisplatin (1 μg/ml) used as a positive control. *means *p* < 0.05, statistical difference.

We first examined miR-4461 expression in OC tissues and normal tissues. The results showed that miR-4461 levels were upregulated in OC tissues compared with normal tissues (Figure 1A). Next, to explore the potential function of miR-4461 in regulating proliferation and metastasis of OC cells, miR-4461 loss-of-function experiments were performed in ovarian cancer cells *in vitro*. HO8910 and A2780 cells were infected with miR-4461 sponge lentivirus and mimic lentivirus. The interference and overexpression effect were confirmed by real-time PCR assay (Figure 1B). OC cell proliferation was quantified using CCK8 assay; the data showed that miR-4461 knockdown suppressed proliferation of OC cells and miR-4461 mimic promoted proliferation of OC cells (Figure 1C). And then, the colony formation assay performed to quantify cell proliferation, the result showed that miR-4461 interference OC cells formed less and smaller colonies (Figure 1D). Data of 5-ethynyl-2′-deoxyuridine (EdU) staining suggested that miR-4461 knockdown inhibited OC cells proliferation and miR-4461 mimic promoted proliferation of OC cells (Figure 1E). Furthermore, flow cytometry data showed a decreased S transition and a marked G0/G1 arrest in miR-4461 knockdown ovarian cancer cells and an increased S transition in miR-4461 mimic ovarian cancer cells (Figures 1F,G). Next, we infected miR-4461 sponge virus in normal ovarian surface epithelial cells (OSE) (Supplementary Figure 1A). The results showed that miR-4461 knockdown did not affect the growth, viability and survival of OSE cells (Supplementary Figures 1B,C). Collectively, miR-4461 promoted ovarian cancer cells proliferation based on the above data.

### miR-4461 Promoted Ovarian Cancer Cells Metastasis *in vitro*

**Figure 2:**
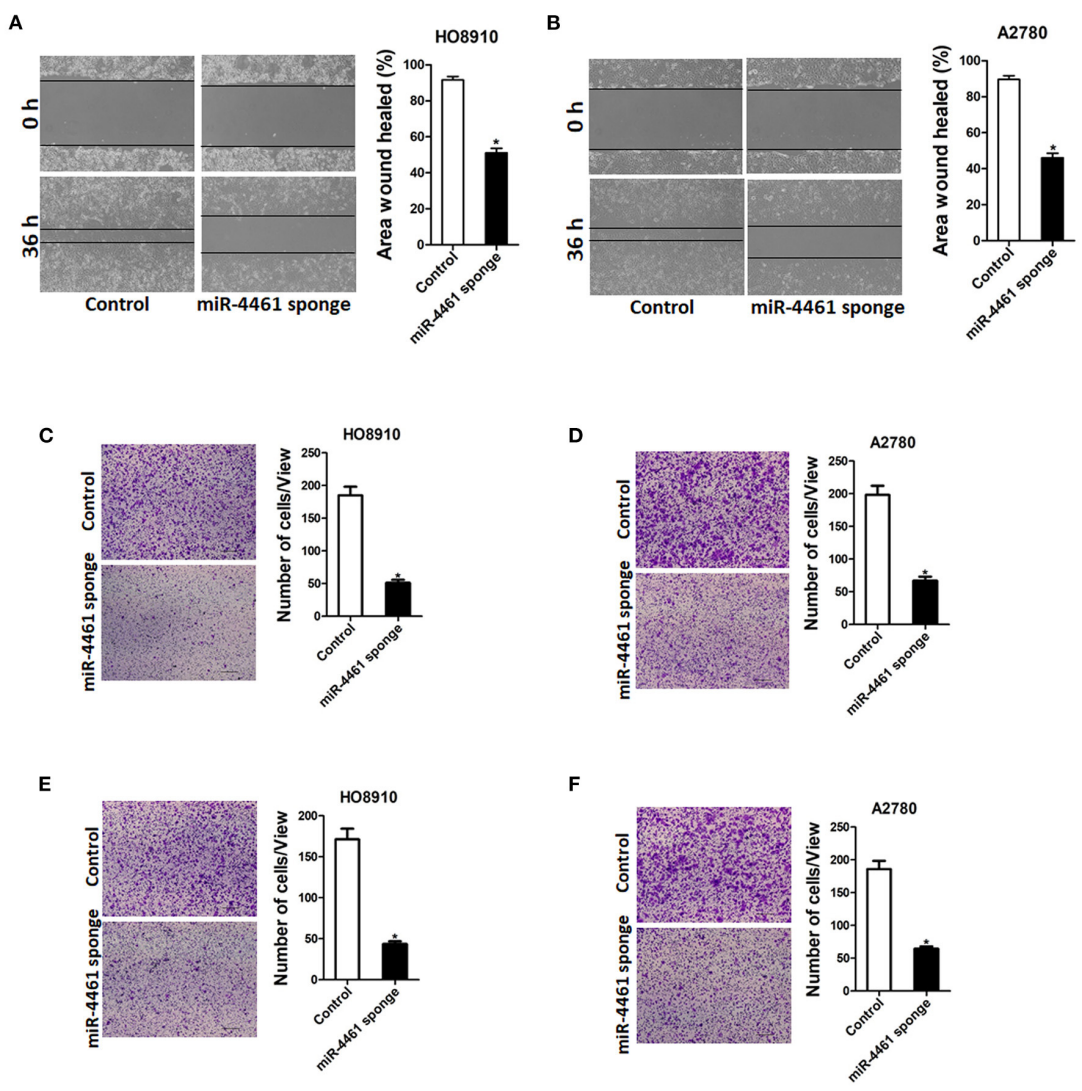
miR-4461 depletion inhibits ovarian cancer cells migration and invasion. **(A)** Wound-healing experiments showed the migration of HO8910 miR-4461 sponge and its control cells. Magnification, 100X; *p < 0.05. **(B)** Wound-healing experiments showed the migration of A2780 miR-4461 sponge and its control cells. Magnification, 100X; *p < 0.05. **(C)** The migration ability of HO8910 miR-4461 sponge and its control cells were measured using polycarbonate membrane inserts in a 24-well plate. **(D)** The migration ability of A2780 miR-4461 sponge and its control cells were measured using polycarbonate membrane inserts in a 24-well plate. **(E)** The invasive capacity of HO8910 miR-4461 sponge and its control cells were quantified *via* Matrigel-coated Boyden chamber. **(F)** The invasive ability of A2780 miR-4461 sponge and its control cells was quantified *via* Matrigel-coated Boyden chamber.

Next, we also explore whether miR-4461 influenced OC cells metastasis. As expected, the wound healing assay results showed that the miR-4461 knockdown ovarian cells had a poorer repairing ability, contrast to the control group (Figures 2A,B). The effect of miR-4461 knockdown on migration and invasion of OC cells was further evaluation by transwell experiments. Consistently, transwell assay revealed that miR-4461 interference impaired the migration ability of OC cells (Figures 2C,D). Matrigel invasion chamber assay showed that the invasion ability was impaired in miR-4461 knockdown ovarian cancer cells (Figures 2E,F). To sum up, our research results indicated that miR-4461 promoted OC cells metastasis.

### miR-4461 Directly Targeted PTEN in Ovarian Cancer Cells

**Figure 3:**
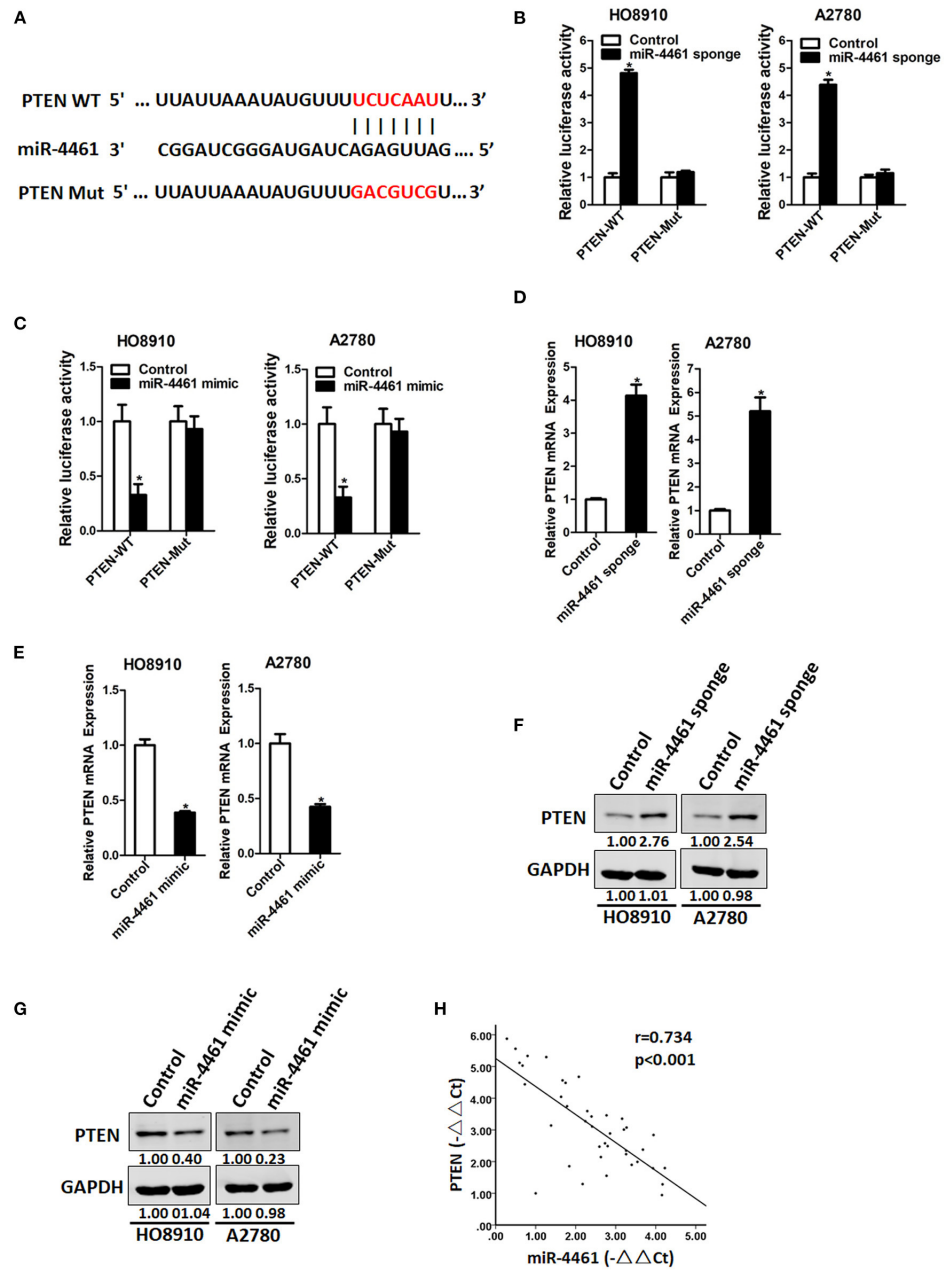
PTEN is a direct target of miR-4461 in ovarian cancer cells. **(A)** A potential target site for miR-4461 in the 3′-UTR of human PTEN mRNA, as predicted by the program Targetscan and miRBase. To disrupt the interaction between miR-4461 and PTEN mRNA, the target site was mutated. **(B)** Luciferase reporter assays performed in HO8910 miR-4461 sponge or A2780 miR-4461 sponge and their control cells transfected with wild-type or mutant PTEN 3′-UTR constructs. **(C)** Luciferase reporter assays performed in HO8910 miR-4461 mimic or A2780 miR-4461 mimic and their control cells transfected with wild-type or mutant PTEN 3′-UTR constructs. **(D)** The mRNA expression of PTEN was determined in HO8910 miR-4461 sponge or A2780 miR-4461 sponge and their control cells by real-time PCR. **(E)** The mRNA expression of PTEN was determined in HO8910 miR-4461 mimic or A2780 miR-4461 mimic and their control cells by real-time PCR. **(F)** The protein expression of PTEN was determined in HO8910 miR-4461 sponge or A2780 miR-4461 sponge and their control cells by western blot. **(G)** The protein expression of PTEN was determined in HO8910 miR-4461 mimic or A2780 miR-4461 mimic and their control cells by western blot. **(H)** Significant correlation was observed between miR-4461 and PTEN expression in human ovarian cancer tissues (*n* = 40). *means *p* < 0.05, statistical difference.

Then, we initiated to seek out the possible target genes of miR-4461 in OC cells. Bioinformatics analysis suggested that miR-4461 has a potential binding site in PTEN mRNA 3′-UTR (Figure 3A). To further identify whether PTEN is the direct target of miR-4461, the wild-type, or mutant PTEN 3′-UTR reporter plasmids were transfected into miR-4461 knockdown or miR-4461 overexpression cells and their control ovarian cancer cells. The results showed that luciferase activity was upregulated by interference of miR-4461 in reporter gene construction containing wild-type 3′-UTR, but not in construction containing mutant 3′UTR (Figure 3B). Conversely, the luciferase activity was decreased by overexpression of miR-4461 in reporter gene construction containing wild-type 3′UTR, but not in construction containing mutant 3′-UTR (Figure 3C). In addition, PTEN mRNA expression was upregulated in miR-4461 knockdown and downregulated in miR-4461 overexpression OC cells (Figures 3D,E). Consistently, PTEN protein expression was also increased in miR-4461 knockdown OC cells and decreased in miR-4461 mimic ovarian cancer cells (Figures 3F,G). A prominent inverse relationship was discovered between miR-4461 and PTEN mRNA expression in human OC tissues (Figure 3H). Collectively, the above results showed that PTEN was a direct target of miR-4461 in OC cells.

### miR-4461 Facilitated Ovarian Cancer Cells Progression via Targeting PTEN

**Figure 4:**
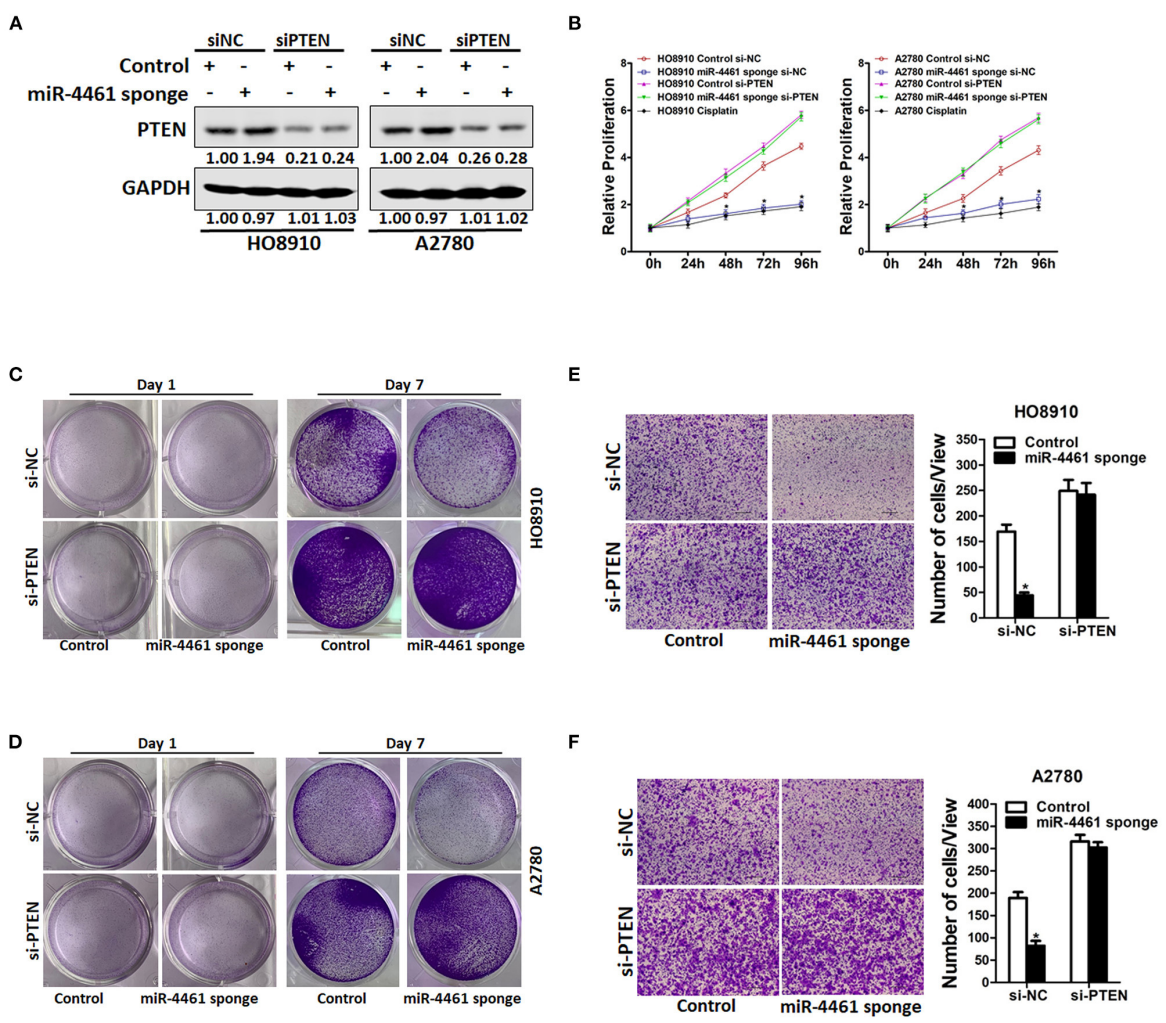
miR-4461 drives ovarian cancer cells proliferation and metastasis *via* targeting PTEN. **(A)** HO8910 miR-4461 sponge or A2780 miR-4461 sponge and their control cells were transfected with si-PTEN or control siRNA and then subjected to western blot assay. **(B)** HO8910 miR-4461 sponge or A2780 miR-4461 sponge and their control cells were transfected with si-PTEN or control siRNA and then subjected to CCK8 assay. Cisplatin (1 μg/ml) used as a positive control. **(C)** HO8910 miR-4461 sponge and its control cells were transfected with si-PTEN or control siRNA and then subjected to colony formation assay. **(D)** A2780 miR-4461 sponge and its control cells were transfected with si-PTEN or control siRNA and then subjected to colony formation assay. **(E)** HO8910 miR-4461 sponge and its control cells were transfected with si-PTEN or control siRNA and then subjected to Invasion assay. **(F)** A2780 miR-4461 sponge and its control cells were transfected with si-PTEN or control siRNA and then subjected to Invasion assay. *means *p* < 0.05, statistical difference.

To further explore the function of PTEN in miR-4461-mediated proliferation and metastasis of ovarian cancer cells, miR-4461 knockdown ovarian cancer cells and control cells was transfected with si-PTEN or control siRNA (Figure 4A). As expected, si-PTEN got rid of the distinct proliferation ability between miR-4461 knockdown OC cells and control cells (Figures 4B–D). Likewise, si-PTEN also eliminated differences of metastasis between miR-4461 knockdown OC cells and their control cells (Figures 4E,F). All in all, the above data suggested that PTEN was involved in miR-4461-mediated OC cells proliferation and metastasis.

### miR-4461 Knockdown Ovarian Cancer Cells Are Sensitive to Cisplatin Treatment

**Figure 5:**
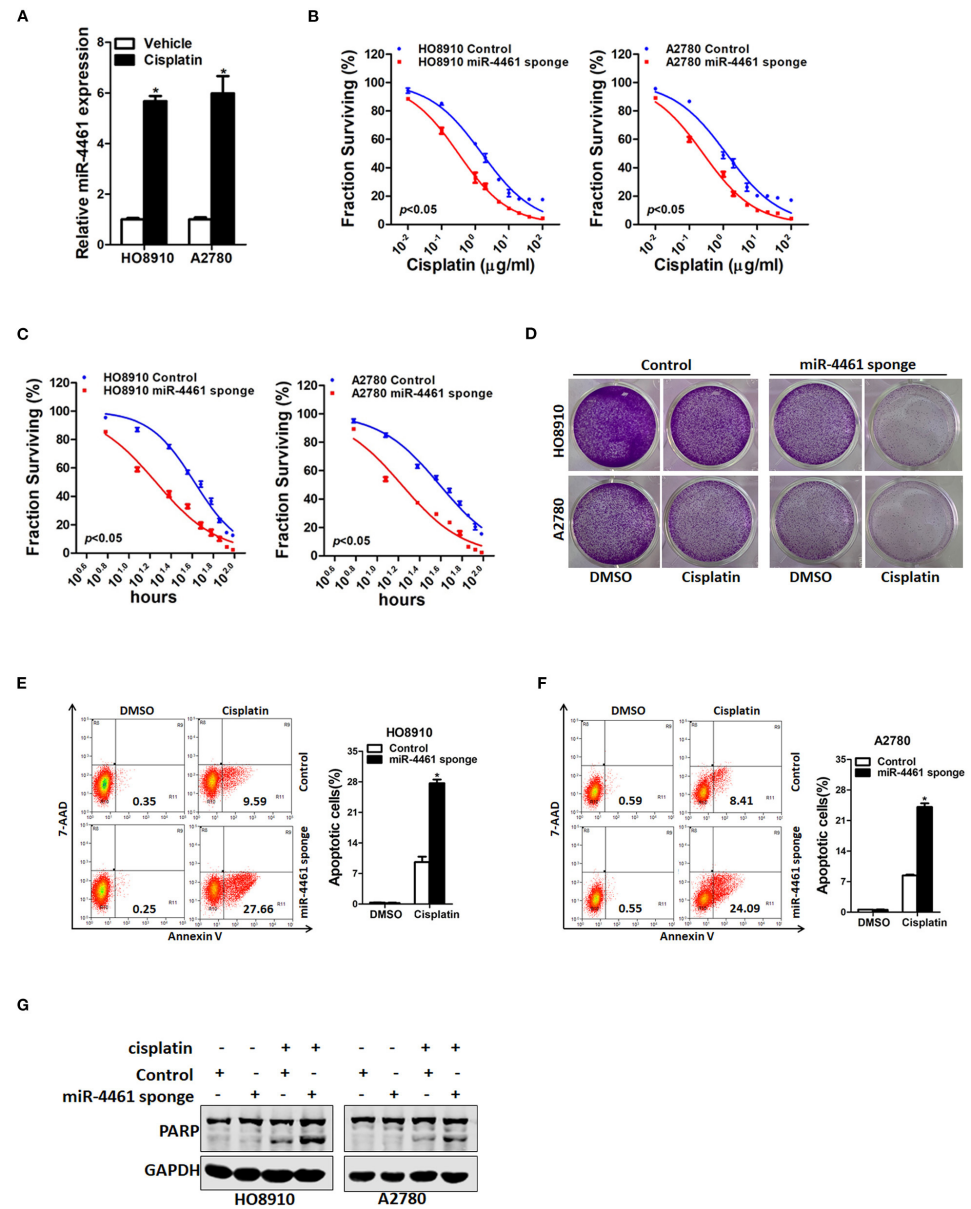
miR-4461 knockdown ovarian cancer cells are sensitive to cisplatin treatment. **(A)** The expression of miR-4461 in cisplatin-resistant ovarian cancer cell lines was measured by real-time PCR. **(B)** Cell survival curves for HO8910 miR-4461 sponge or A2780 miR-4461 sponge and their control cells treated cisplatin with a dose escalation from 0 to 100 μg/ml. Data are presented as mean ± SD (*n* = 4) from one of three independent experiments. **(C)** Cell survival curves for HO8910 miR-4461 sponge or A2780 miR-4461 sponge and their control cells treated cisplatin (4 μg/ml) from 0 to 96 h. Data are presented as mean ± SD (*n* = 4) from one of three independent experiments. **(D)** HO8910 miR-4461 sponge or A2780 miR-4461 sponge and their control cells were treated with cisplatin (1 μg/ml) for 7 days and their colony growth was examined. **(E)** HO8910 miR-4461 sponge and its control cells were treated with cisplatin (4 μg/ml) for 48 h. The apoptotic cells were detected by flow cytometry. (7-AAD negative and Annexin V positive cells are the apoptotic cells). **(F)** A2780 miR-4461 sponge and its control cells were treated with cisplatin (4 μg/ml) for 48 h. The apoptotic cells were detected by flow cytometry. (7-AAD negative and Annexin V positive cells are the apoptotic cells). **(G)** HO8910 miR-4461 sponge or A2780 miR-4461 sponge and their control cells were treated with cisplatin (4 μg/ml) for 48 h. The cell extracts were subjected to western blotting with special antibody against PARP. *means *p* < 0.05, statistical difference.

We next investigated whether miR-4461 participated regulation of chemo-resistance of OC cells. We first measured the expression of miR-4461 in cisplatin-resistance OC cells. The data suggested that miR-4461 expression was dramatically increased in cisplatin-resistance OC cells (Figure 5A). In addition, the sensitivity of cisplatin was upregulated in miR-4461 knockdown OC cells contrasted to control cells (Figures 5B,C). Data also showed that miR-4461 knockdown led to the sensitive of OC cells to cisplatin-induced growth suspension (Figure 5D). Consistently, interference of miR-4461 sensitized ovarian cancer cells to undergo cisplatin-induced cell apoptosis (Figures 5E–G). Collectively, the results demonstrated that miR-4461 interference ovarian cancer cells are more sensitive to cisplatin treatment.

## Discussion

Increasing evidence demonstrated that miRNAs are important regulators in ovarian cancer, however, few miRNAs have been used clinically. Earlier studies pointed out that miR-4461 was a tumor suppressor in renal cell carcinoma and colorectal cancer (13, 14). We found that interference of miR-4461 expression in OC cells suppressed cell proliferation and metastasis. PTEN, a tumor suppressive gene, was identified as a direct target gene of miR-4461 in OC cells. Our research for the first time confirmed that miR-4461 promoted OC cell growth and metastasis by targeting PTEN, which points out that miR-4461 functions as an oncomiRNA.

miR-4461 is a newfound miRNA, its functional features and mechanism in biological processes and diseases are not fully understood. Previous studies showed that miR-4461 inhibits tumorigenesis of renal cell carcinoma and colorectal cancer. However, whether miR-4461 is involved in ovarian cancer remains unknown. This study demonstrated that miR-4461 can significantly accelerate the proliferation and metastasis of OC cells. Moreover, miR-4461 can downregulate PTEN protein expression by directly targeting PTEN 3′-UTR.

PTEN is an important tumor suppressor, and has been found to participate in the tumorigenesis and progression *via* downregulation of PI3-K/Akt pathway (18–20). PTEN was a vital regulator of cell reproduction, cell cycle, apoptosis and metastasis, and its low expression might cause tumor progression including OC (21–23). In our research, we demonstrated that PTEN was not only a downstream target gene of miR-4461, but also a functional medium of miR-4461 in OC cells. And a complementary sequence of miR-9 in the 3′-UTR of PTEN was confirmed by luciferase assay. The luciferase activity of wide type PTEN 3′-UTR was suppressed, but there was no effect on in the mutant PTEN 3′-UTR when OC cells were transfected with miR-4461 mimic or the wide type of PTEN 3′-UTR. On the contrary, the luciferase activity of wide type PTEN 3′-UTR was increased, but there was no effect on in the mutant PTEN 3′-UTR when OC cells were transfected with miR-4461 sponge or the wide type of PTEN 3′-UTR. It implied that miR-4461 might be combined with PTEN 3′-UTR and then inhibited PTEN transcription. Further study indicated that miR-4461 knockdown enhanced PTEN mRNA and protein expression in OC cells. Instead, miR-4461 overexpression suppressed PTEN mRNA and protein expression in OC cells. miR-4461 expression was negatively associated with PTEN expression in OC tissues. Moreover, PTEN siRNA could eliminate the distinct proliferation capacity or metastasis ability between miR-4461 overexpression OC cells and control cells.

Acquired cisplatin resistance is a serious problem for the therapy of OC patients (8, 24). miRNAs actually participated in the regulation of tumorigenesis and drug resistance in OC (11, 25). By quantified the expression of miR-4461 in cisplatin-resistant cell lines, we found that miR-4461 expression was enhanced in resistant cells, suggesting that high expression of miR-4461 might be associated with cisplatin resistance in OC. In addition, the potential clinical utility of miR-4461 was investigate through the loss-of-function experiments. MTT analysis showed that miR-4461 knockdown descended the IC50 of cisplatin and cell viability in cisplatin-resistant cells. Furthermore, the result of flow cytometry as well as expression of apoptotic protein revealed that miR-4461 knockdown promoted cell apoptosis of cisplatin-resistant cells. These results uncovered the sensitive effect of miR-4461 knockdown on cisplatin resistance in OC *in vitro*.

In conclusion, our finding revealed that miR-4461 promote ovarian cancer proliferation and metastasis *via* directly regulating PTEN. Moreover, we demonstrated that miR-4461 is effective in determining cisplatin response in ovarian cancer. These findings of the present study not only shed a new light on the mechanism of ovarian cancer but suggest a potential therapeutic target against ovarian cancer patients.

## Data Availability Statement

The raw data supporting the conclusions of this article will be made available by the authors, without undue reservation.

## Ethics Statement

The studies involving human participants were reviewed and approved by the Clinical Research Ethics Committees of The First Affiliated Hospital of China Medical University. Written informed consent to participate in this study was provided by the participants' legal guardian/next of kin. Written informed consent was obtained from the individual(s), and minor(s)' legal guardian/next of kin, for the publication of any potentially identifiable images or data included in this article.

## Author Contributions

LD and YZ conducted all experiments and analyzed the data. LD provided clinical samples, provided pathology evaluation, analyzed clinical data, and wrote the manuscript. YZ provided support with experimental techniques, contributed to the revision, conceived the project, and supervised all experiments.

## Conflict of Interest

The authors declare that the research was conducted in the absence of any commercial or financial relationships that could be construed as a potential conflict of interest.
