# Supplementary material for: miR-4461 Regulates the Proliferation and Metastasis of Ovarian Cancer Cells and Cisplatin Resistance
Source: Front Oncol. 2021 Mar 9;11:614035. doi: 10.3389/fonc.2021.614035 (PMC7985457; doi:10.3389/fonc.2021.614035)
Supplement: Supplementary file 1 [file Table_1.DOCX]

**miR-4461 regulates the proliferation and metastasis of** **ovarian cancer cells and cisplatin resistance**

Lei Dou^1^, Yi Zhang ^1,*^

^1^Department of Gynecology, the First Affiliated Hospital of China Medical University, Shenyang 110001, Liaoning, China.

Corresponding authors: Dr. Yi Zhang, Department of Gynecology, The First Affiliated Hospital of China Medical University, Shenyang 110001, China. Email address: zhangyi@cmu1h.com.

**Running title:** miR-4461 drives ovarian cancer cells progression.


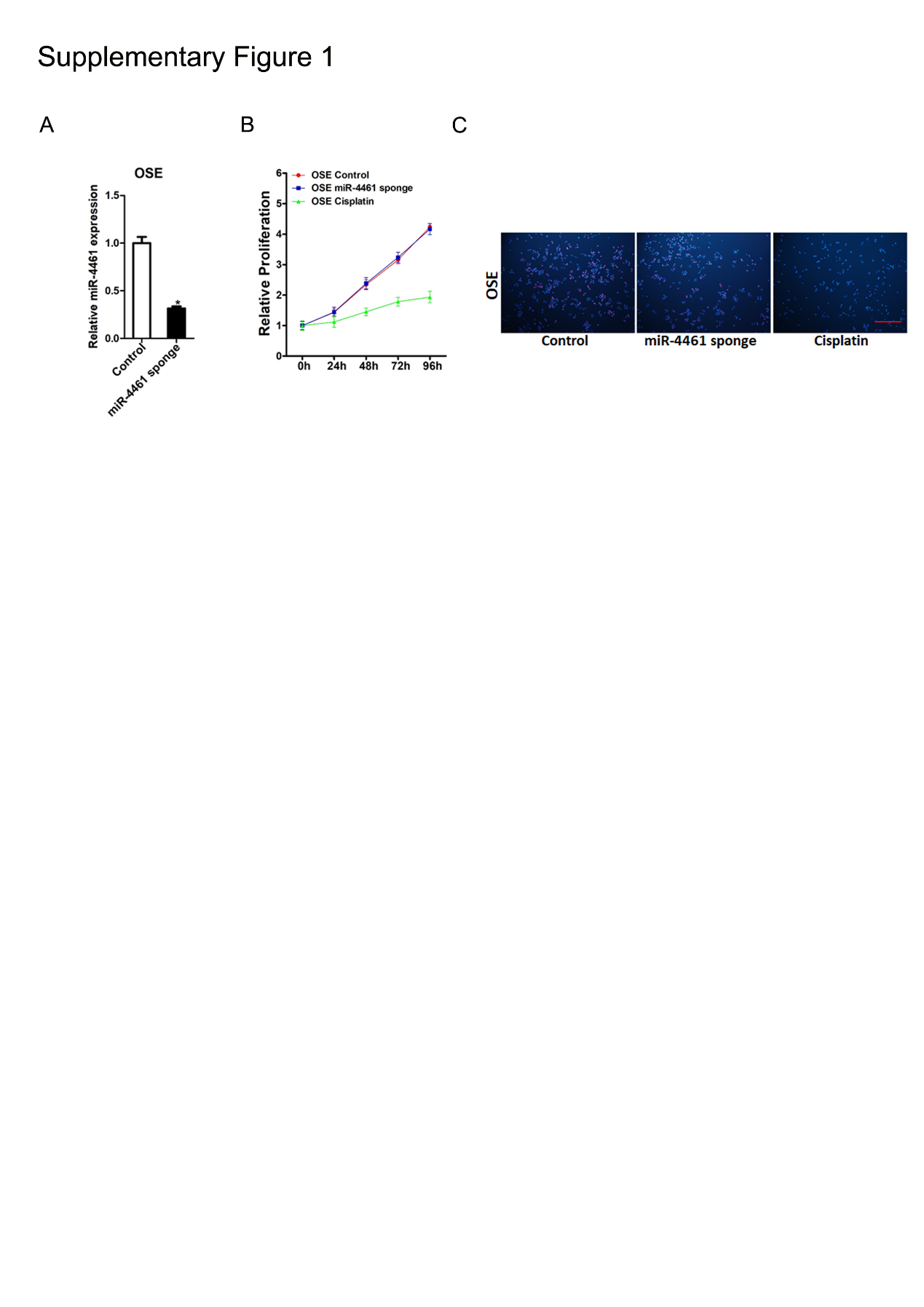


**Supplementary Figure1 Legends**

A. The knockdown and overexpression effect of miR-4461 in OSE cells was detected by real-time PCR analysis.

B. Cell proliferation in OSE miR-4461 sponge and its control cells was quantified by using CCK-8 assays. Cisplatin (2 μg/ml) used as a positive control.

C. Representative images of EdU staining of proliferating OSE miR-4461 sponge and its control cells. EdU+ cells were stained with red immunofluorescence. The nuclei were counterstained with DAPI (blue). Cisplatin (2 μg/ml) used as a positive control. Scale bar=50 μm.
