# Supplementary figures and images for: miR-4461 Regulates the Proliferation and Metastasis of Ovarian Cancer Cells and Cisplatin Resistance
Source: Front Oncol. 2021 Mar 9;11:614035. doi: 10.3389/fonc.2021.614035 (PMC7985457; doi:10.3389/fonc.2021.614035)

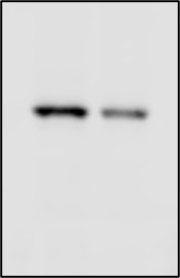

Supplement: Supplementary file 2 [file Data_Sheet_1.ZIP › WB/Figure 3F-1.tif]

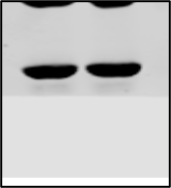

Supplement: Supplementary file 2 [file Data_Sheet_1.ZIP › WB/Figure 3F-2.tif]

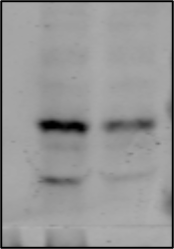

Supplement: Supplementary file 2 [file Data_Sheet_1.ZIP › WB/Figure 3F-3.tif]

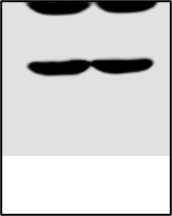

Supplement: Supplementary file 2 [file Data_Sheet_1.ZIP › WB/Figure 3F-4.tif]

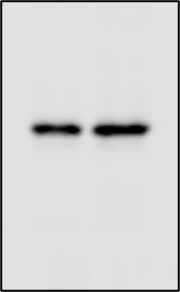

Supplement: Supplementary file 2 [file Data_Sheet_1.ZIP › WB/Figure 3G-1.tif]

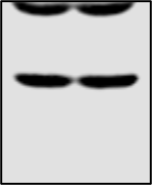

Supplement: Supplementary file 2 [file Data_Sheet_1.ZIP › WB/Figure 3G-2.tif]

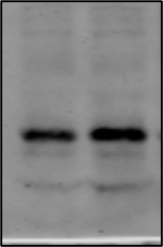

Supplement: Supplementary file 2 [file Data_Sheet_1.ZIP › WB/Figure 3G-3.tif]

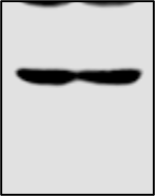

Supplement: Supplementary file 2 [file Data_Sheet_1.ZIP › WB/Figure 3G-4.tif]

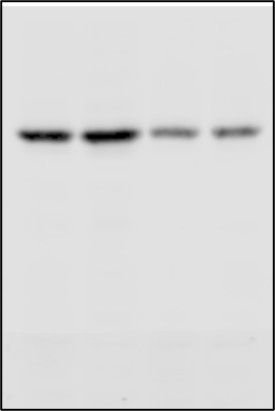

Supplement: Supplementary file 2 [file Data_Sheet_1.ZIP › WB/Figure 4A-1.tif]

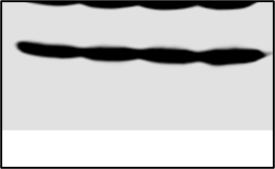

Supplement: Supplementary file 2 [file Data_Sheet_1.ZIP › WB/Figure 4A-2.tif]

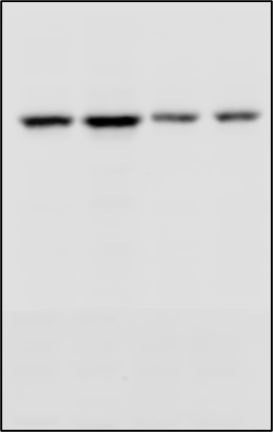

Supplement: Supplementary file 2 [file Data_Sheet_1.ZIP › WB/Figure 4A-3.tif]

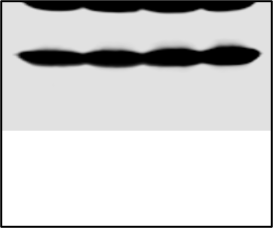

Supplement: Supplementary file 2 [file Data_Sheet_1.ZIP › WB/Figure 4A-4.tif]

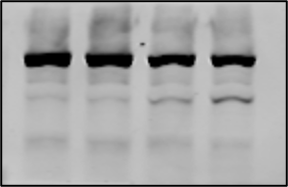

Supplement: Supplementary file 2 [file Data_Sheet_1.ZIP › WB/Figure 5G-1.tif]

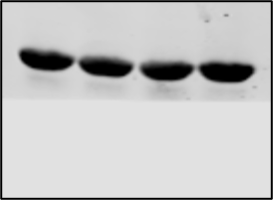

Supplement: Supplementary file 2 [file Data_Sheet_1.ZIP › WB/Figure 5G-2.tif]

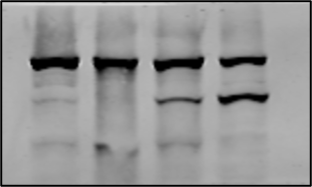

Supplement: Supplementary file 2 [file Data_Sheet_1.ZIP › WB/Figure 5G-3.tif]

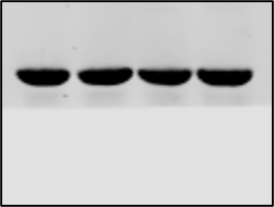

Supplement: Supplementary file 2 [file Data_Sheet_1.ZIP › WB/Figure 5G-4.tif]

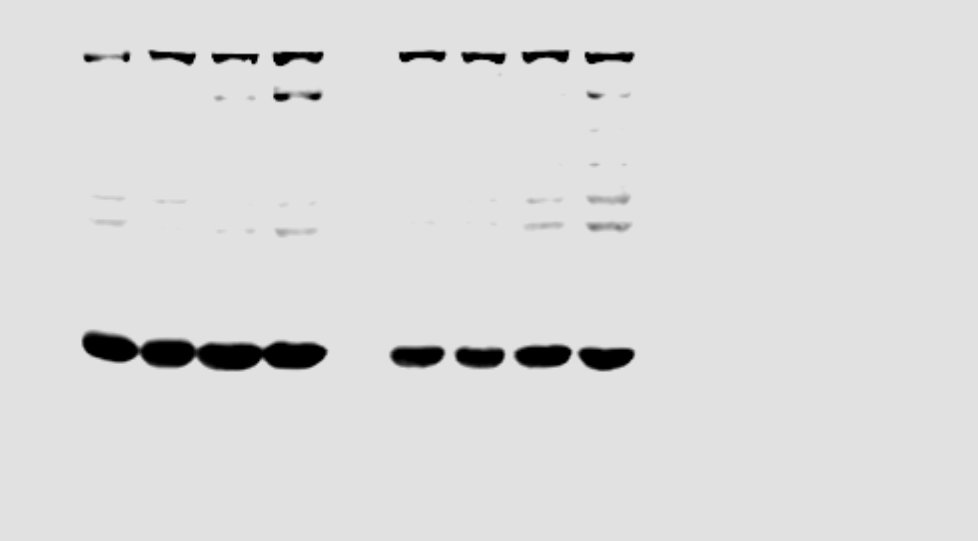

Supplement: Supplementary file 3 [file Data_Sheet_2.ZIP › WB-2/Image_GAPDH-2.tif]

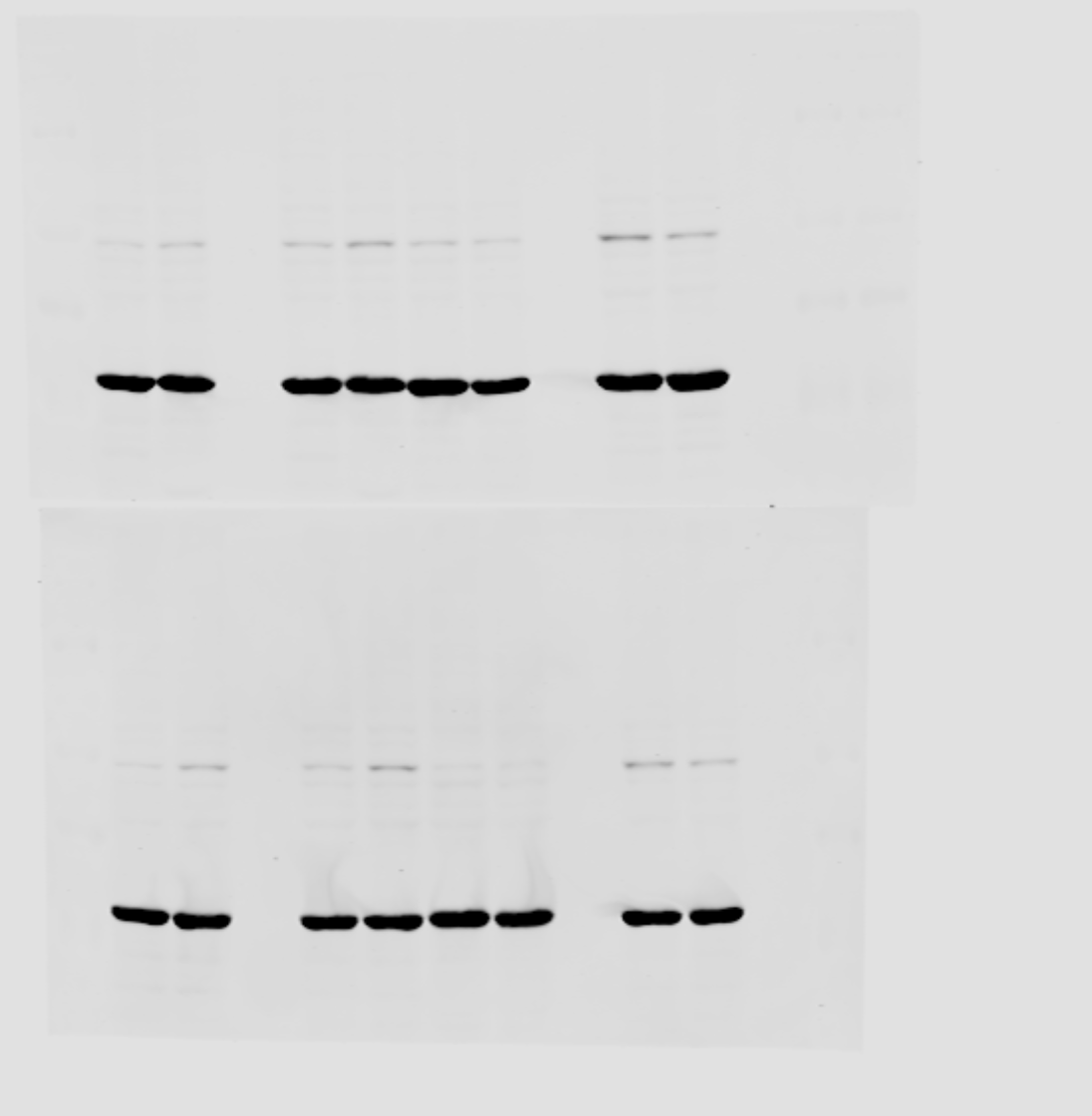

Supplement: Supplementary file 3 [file Data_Sheet_2.ZIP › WB-2/Image_GAPDH.tif]

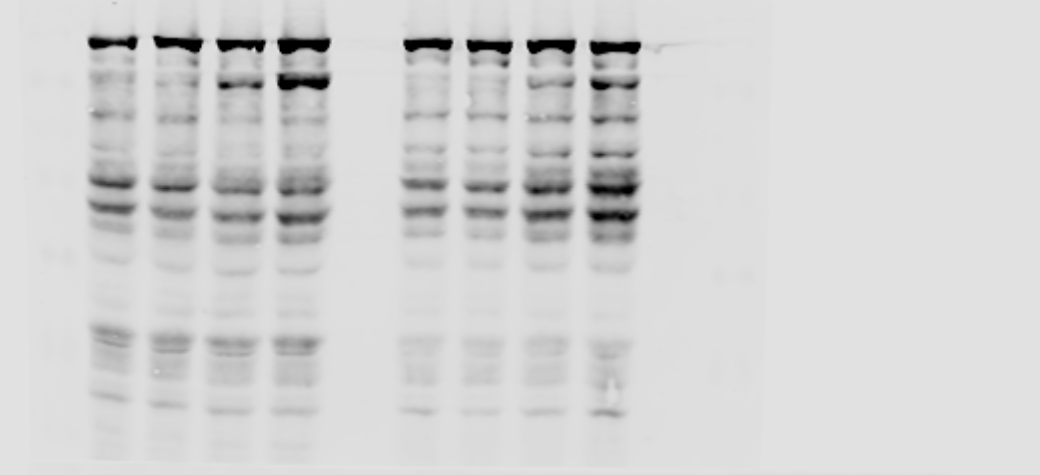

Supplement: Supplementary file 3 [file Data_Sheet_2.ZIP › WB-2/Image_PARP.tif]

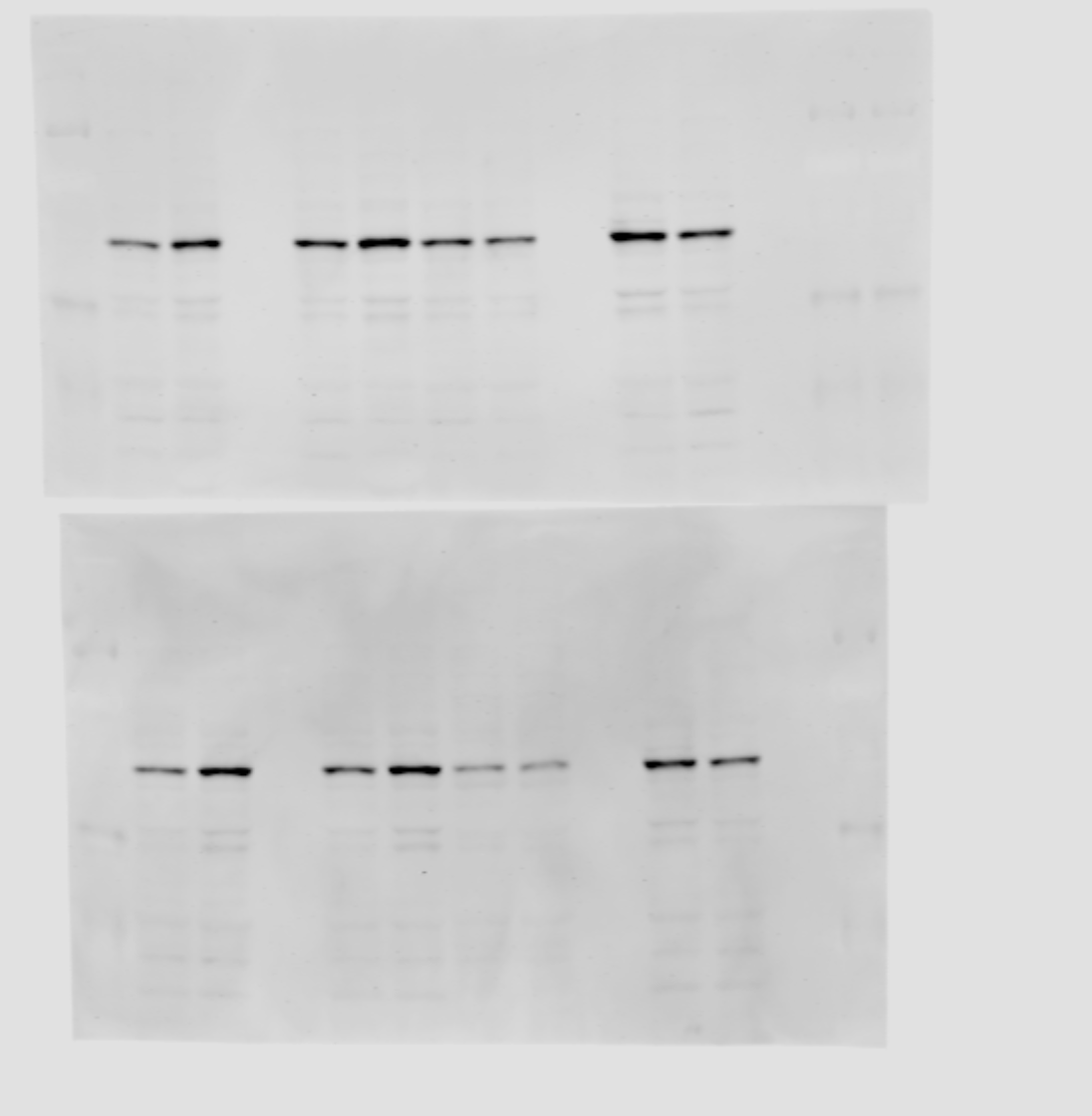

Supplement: Supplementary file 3 [file Data_Sheet_2.ZIP › WB-2/Image_PTEN.tif]
